# Metabolic Response of *Pleurotus ostreatus* to Continuous Heat Stress

**DOI:** 10.3389/fmicb.2019.03148

**Published:** 2020-01-21

**Authors:** Zhiyu Yan, Mengran Zhao, Xiangli Wu, Jinxia Zhang

**Affiliations:** ^1^Institute of Agricultural Resources and Regional Planning, Chinese Academy of Agricultural Sciences, Beijing, China; ^2^Key Laboratory of Microbial Resources, Ministry of Agriculture and Rural Affairs, Beijing, China

**Keywords:** *Pleurotus ostreatus*, heat stress, temporality, intracellular, metabolic response

## Abstract

Heat stress seriously threatens the growth of *Pleurotus ostreatus*. Various studies have been performed to study the resistance of *P. ostreatus* to heat stress. Here, the metabolome was evaluated to determine the response of *P. ostreatus* mycelia to heat stress at different times (6, 12, 24, 48 h). More than 70 differential metabolites were detected and enriched in their metabolic pathways. Dynamic metabolites changes in enrichment pathways under heat stress showed that heat stress enhanced the degradation of unsaturated fatty acids and nucleotides, increased the content of amino acids and vitamins, and accelerated glycolysis and the tricarboxylic acid cycle in *P. ostreatus*. The time course changes of *P. ostreatus* metabolites under continuous heat stress demonstrated that amino acids continuously changed with heat stress, nucleotides clearly changed with heat stress at 12 and 48 h, and lipids exhibited an increasing trend with prolonged heat stress, while few types saccharides and vitamins changed under heat stress. Additionally, heat-treated *P. ostreatus* produced salicylic acid and other stress-resistant substances that were reported in plants. This study first reported the metabolites changes in *P. ostreatus* mycelia during 48 h of heat stress. The metabolic pathways and substances that changed with heat stress in this research will aid future studies on the resistance of *P. ostreatus* and other edible fungi to heat stress.

## Introduction

*Pleurotus ostreatus* has economic and ecological values and medicinal properties and has thus been intensely studied and cultivated in many different areas of the world (Sánchez, 2010). However, the cultivation of *P. ostreatus* is still dependent on the natural environmental temperature in China and many other developing countries (Lei et al., 2019). Thus, high temperature is the most adverse stress in the cultivation of *P. ostreatus*. After high-temperature stress, mycelial growth is inhibited, mycelia are susceptible to infection by pathogens, such as *Trichoderma asperellum*, and apoptotic-like cell death can occur (Song et al., 2014; Qiu et al., 2017). These adverse effects cause enormous economic losses, which urgently call for studies on the mechanism of *P. ostreatus* resistance to heat stress.

Fortunately, a few correlative studies have been performed on some edible and medicinal fungi. First, relevant reports on heat shock proteins (HSPs) are abundant. Zou et al. (2018) found that the expression of some HSPs increased under heat stress, and the expression of HSPs was also upregulated in *Ganoderma lucidum* (Zhang et al., 2016). Second, cell signals were involved in the heat stress response. Kong et al. (2012) discovered that nitric oxide (NO) can alleviate heat stress-induced oxidative damage in *Pleurotus eryngii*. Other signaling molecules similar to NO, such as calcium (Ca^2+^), reactive oxygen species (ROS), and hydrogen sulfide (H_2_S), were found to participate in the heat stress response in *G. lucidum* (Zhang et al., 2016; Liu et al., 2018b; Tian et al., 2019). Finally, further studies have reported activated phospholipase D (PLD), a significant accumulation of phosphatidic acid (PA) (Liu et al., 2017a), and increased cell membrane fluidity upon heat stress (Liu et al., 2017b). These studies analyzed the mechanisms of the edible and medicinal fungal response to heat stress at different levels, and yet, it remains unclear whether there are other heat-response strategies.

Coincidentally, metabolic change is generally a mechanism by which biology copes with stress. Except for reports on animals and plants (Baumgard and Rhoads, 2013; Victoria Sanz Fernandez et al., 2015; Narayanan et al., 2016; Das et al., 2017), these metabolic changes are most common in yeast. *Saccharomyces cerevisiae* reprograms non-fermentative metabolism via stress-responsive transcription factors (Soontorngun, 2017), further changes intracellular metabolism underlying ethanol stress (Tian et al., 2017), and increases the intermediates in some metabolic pathways when experiencing oxidative stress (Boone et al., 2017). However, whether metabolic changes occur in edible and medicinal fungi under heat stress remains unclear, but such changes may play a significant role in understanding the mechanism of the response of *P. ostreatus* to heat stress.

In this study, the metabolome was evaluated to detect the metabolite dynamics in *P. ostreatus* mycelia under continuous heat stress to clarify the metabolic response of *P. ostreatus* to heat stress, thus aiding heat tolerance research of *P. ostreatus* and other edible and medicinal fungi.

## Materials and Methods

### Strains

*Pleurotus ostreatus* 389 (CCMSSC 00389), obtained from the China Center for Mushroom Spawn Standards and Control, was used in this study.

### Culture Conditions, Heat Treatment and Sample Collection

An activated mycelial block of *P. ostreatus* (7 mm) was inoculated on complete yeast medium (CYM) containing 1% maltose, 2% glucose, 0.2% peptone, 0.2% yeast extract, 0.05% MgSO_4_⋅7H_2_O, and 0.46% KH_2_PO_4_ with an initial pH of 5.5 (Wang et al., 2018). The plates were cultured at 28°C in the dark for 5 days before use.

Heat treatment began on the sixth day at 40°C (Lei et al., 2019) for 0–48 h, and after heat treated 48 h, the mycelia were recovered for 6 h at 28°C. All treatments included controls at 28°C. The samples were collected in tubes after heat treatment for 0, 6, 12, 24, 48 h (Wang, 2019), and 48 h + 6 h (means heat treated 48 h and then recovered 6 h) with seven repeats per group. The tubes were frozen in liquid nitrogen as soon as samples were collected and finally stored at −70°C until use.

### Basic Growth Phenotype Measurements of *P. ostreatus*

To evaluate the effect of heat stress on *P. ostreatus*, the mycelial fresh weight, and macroscopic and microscopic alteraons in the mycelia, contents of total carbohydrate and total protein were measured. Once the mycelia begin with heat treatment, take photos for each treated mycelium to record the growth status, and measured the fresh weight after sample collection. Microscopic changes of mycelia were detected using a Zeiss Axio Imager A1 fluorescence microscope and analyzed using Zeiss software (ZEN lite, Zeiss, Göttingen, Germany). The contents of total carbohydrate and total protein were measured using commercial kits, Total Carbohydrate Assay Kit (Solarbio Life Sciences) and Biuret Protein Assay Kit (Solarbio Life Sciences), according to manufacturer directions.

### Metabolomic Analysis of *P. ostreatus* Mycelia by Liquid Chromatography-Mass Spectrometry (LC-MS)

Metabolomic analysis was performed on the samples described above by LC-MS according to previously described methods (Xue et al., 2017) with modifications. The freeze-dried samples were extracted with methanol and an internal standard at a ratio of 500:8:0.1. All samples were ground to a fine powder using a grinding mill at 65 Hz for 90 s. Then, the samples were ultrasonicated for 30 min at 40 KHz and allowed to stand for 1 h at −20°C. The samples were centrifuged at 12000 rpm at 4°C for 15 min, and 200 μL of supernatant was transferred to vials for LC-MS analysis.

The analysis platform was LC-Q/TOF-MS (Agilent, 1290 Infinity LC, 6530 UHD and Accurate-Mass Q-TOF/MS, Agilent Technologies, Santa Clara, CA, United States) with a Waters column (ACQUITY UPLC@HSS T3, 2.5 μm 100^∗^2.1 mm). Water with 0.1% formic acid and acetonitrile with 0.1% formic acid were used for mobile phase A and B, respectively; the flow rate was 0.4 mL/min, the injection volume was 4 μL, the automatic injector temperature was 4°C, and the column temperature was 40°C. The capillary voltage was 4 and 3.5 kV in positive and negative mode, respectively; the drying gas flow was 11 L/min, and the gas temperature was 350°C. Centroid data were collected from 100 to 1000 m/z.

### Statistical Analysis and Pathway Construction

The tests of basic growth phenotype measurements were three biological repeats at least and data are presented as mean ± SD. Statistical significance was defined as *p* < 0.05. The statistical analysis was performed using Graphpad Prism 6, Stst and Excel 2010 software.

The data obtained from the metabolom were subjected to feature extraction and preprocessed with XCMS in R software and then normalized and edited into a two-dimensional data matrix using Excel 2010 software. SIMCA-P software was used for multivariate statistical analyses, which included principal component analysis (PCA), partial least squares-discriminant analysis (PLS-DA), and orthogonal partial least squares-discriminant analysis (OPLS-DA). The differential metabolites were confirmed by the combination of variable importance in the projection (VIP) value (VIP > 1) from the OPLS-DA model and the *p*-value (*p* < 0.05) from Student’s *t*-test. The identity of differential metabolites was determined by searching online databases (Metlin) and comparing their m/z and mass. The metabolomics analysis was carried out by Shanghai Sensichip Infotech Co. (Shanghai, China).

All metabolomic tests included seven biological repeats, and the data are presented as the mean after normalizing (Lee et al., 2017). The fold change of the different substances was the ratio of the value at 40°C to that at 28°C. The statistical analyses were performed using GraphPad Prism 6 and Excel 2010 software. The pathway analyses of differential substances were performed with MetaboAnalyst 4.0.

## Results

### The Effect of Heat Stress on *P. ostreatus*

High temperature is a severe stress to *P. ostreatus*. Once the mycelia suffered from heat stress, the biomass (fresh weight) were almost unchanged as shown in Supplementary Figure S1A, when comparing with the control, the biomass of mycelia decreased significantly after 48 h of heat stress. A short-term recovery (6 h) after heat stress did not affect the biomass of mycelia. Macroscopically, the aerial mycelia in normal growth conditions grew fast and flourishingly, and became loosely and wiltingly after heat stress, almost ceased growing (Supplementary Figure S1B). From microscopical aspect (Supplementary Figure S1C), the mycelia in control group were thick and vigorous; after heat stress, the mycelia were distorted, easily broken and overlapped. And heat stress can make the total protein content of mycelia reduced significantly (Supplementary Figure S1D), which was consistent with the inhibition of heat stress on the mycelia biomass; besides, after 24 h of heat stress, the total carbohydrate content of mycelia also reduced significantly (Supplementary Figure S1E).

**FIGURE 1:**
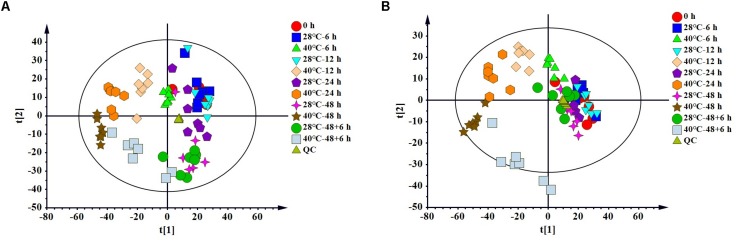
The PCA score plot of LC-MS data of all *P. ostreatus* samples. **(A)** The positive mode, **(B)** the negative mode.

To clarify the changes in intracellular metabolites of *P. ostreatus* in response to heat stress, metabolic changes between non-heat-stressed (28°C) and heat-stressed (40°C) samples were detected using LC-MS. A total of 1805 features were detected in positive mode, and 1621 features were detected in negative mode. Multivariate statistical analysis of normalized data was performed. The PCA score plot of all samples demonstrated that a total of 14 principal components were obtained in the positive (*R*^2^X = 0.827, *Q*^2^ = 0.652) and negative (*R*^2^X = 0.836, *Q*^2^ = 0.671) modes, which indicated a clear difference between the 28°C and 40°C groups (Figure 1). To distinguish the differences between the 28°C and 40°C groups, PLS-DA and OPLS-DA were subsequently used in the 10 data analyses (Table 1). The results showed that each model was reliable for explaining the differences between the two groups and obtaining the different substances, and there were no “overfitting” phenomena in the models according to the ranking validation chart, suggesting the availability of these data for subsequent analyses.

**TABLE 1:** The PLS-DA and OPLS-DA scatter plots of LC-MS data of *P. ostreatus* samples in each of the two groups.

Groups	PLS-DA						OPLS-DA					
	Positive mode			Negative mode			Positive mode			Negative mode		
	*R*^2^X	*R*^2^Y	*Q*^2^	*R*^2^X	*R*^2^Y	*Q*^2^	*R*^2^X	*R*^2^Y	*Q*^2^	*R*^2^X	*R*^2^Y	*Q*^2^
A	0.519	0.993	0.938	0.664	0.998	0.971	0.643	1.000	0.972	0.756	0.999	0.958
B	0.556	0.994	0.957	0.588	0.986	0.910	0.556	0.994	0.949	0.588	0.986	0.922
C	0.610	0.998	0.982	0.654	0.996	0.979	0.610	0.998	0.978	0.654	0.996	0.972
D	0.642	0.997	0.963	0.673	0.996	0.949	0.642	0.997	0.968	0.673	0.996	0.942
E	0.617	0.997	0.969	0.631	0.991	0.958	0.617	0.997	0.967	0.722	1.000	0.968
F	0.521	0.995	0.962	0.613	0.997	0.980	0.521	0.995	0.968	0.613	0.997	0.977
G	0.531	0.992	0.946	0.568	0.995	0.971	0.531	0.992	0.959	0.568	0.995	0.966
H	0.599	0.992	0.962	0.607	0.997	0.963	0.599	0.992	0.955	0.607	0.997	0.962
I	0.599	0.997	0.964	0.666	0.999	0.996	0.599	0.997	0.962	0.666	0.999	0.992
J	0.592	0.990	0.956	0.587	0.990	0.965	0.592	0.990	0.953	0.587	0.990	0.956

*The letters in the groups represent each of two groups comparisons as follows: A: 40°C, 6 h–0 h; B: 40°C, 12 h–0 h; C: 40°C, 24 h–0 h; D: 40°C, 48 h–0 h; E: 40°C, 48 h + 6 h–0 h; F: 40°C, 6 h–28°C, 6 h; G: 40°C, 12 h–28°C, 12 h; H: 40°C, 24 h–28°C, 24 h; I: 40°C, 48 h–28°C, 48 h; J: 40°C, 48 h + 6 h–28°C, 48 h + 6 h (48 h + 6 h means heat treated 48 h and then recovered 6 h, the same below). The model quality of both PLS-DA and OPLS-DA were discriminated by *R*^2^Y and *Q*^2^ values, and *R*^2^Y > 0.5 and *Q*^2^ > 0.5 indicated a good model; values closer to 1 indicated a better model.*

### Metabolic Changes Under Heat Stress and the Metabolic Pathway Enrichment of Responsive Metabolites

**FIGURE 2:**
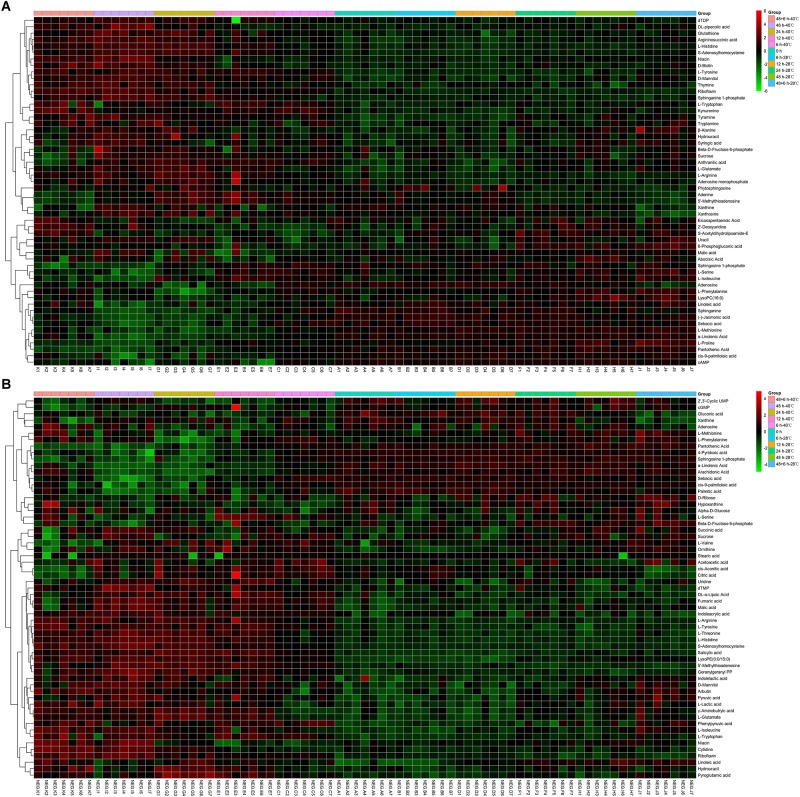
Heatmap analysis of the diverse metabolites in *P. ostreatus* samples with or without heat treatment. **(A)** The positive mode, **(B)** the negative mode.

Differential metabolites were identified by a statistically significant analysis with *p* < 0.05 and VIP > 1. There were 54 metabolites with significant changes in the positive mode and 59 metabolites with significant changes in the negative mode (Figure 2). Moreover, to further understand the effect of heat stress on the mycelial metabolism of *P. ostreatus*, all differential metabolites in each group were calculated by MetaboAnalyst 4.0 to enrich the pathways to which the metabolites belonged. Among these, the data for the sample 40°C 6 h–0 h were chosen randomly and are shown in Table 2; 32 pathways were identified. Among these, the metabolic pathways of significant changes under heat stress mainly include glutathione metabolism, sphingolipid metabolism and some amino acids metabolism.

**TABLE 2:** The enrichment pathways of altered metabolites at 40°C, 6 h–0 h.

No.	Pathway name	Match status	*p*	Impact	Involved metabolites
1	Citrate cycle (TCA cycle)	5/20	0.004	0.304	Pyruvic acid, fumaric acid, citric acid, *cis*-aconitic acid, *S*-acetyldihydrolipoamide-E
2	Phenylalanine, tyrosine and tryptophan biosynthesis	5/22	0.006	0.147	L-tryptophan, L-phenylalanine, L-tyrosine, anthranilic acid, Phenylpyruvic acid
3	Aminoacyl-tRNA biosynthesis	8/67	0.031	0.093	L-arginine, L-serine, L-methionine, L-tryptophan, L-phenylalanine, L-tyrosine, LHistidine, L-Isoleucine
4	Cysteine and methionine metabolism	5/33	0.035	0.166	*S*-adenosylhomocysteine, pyruvic acid, L-serine, L-methionine, 5′-methylthioadenosine
5	Glycolysis or Gluconeogenesis	4/24	0.043	0.216	Pyruvic acid, alpha-D-glucose, beta-D-fructose 6-phosphate, *S*-acetyldihydrolipoamide-E
6	Phenylalanine metabolism	2/7	0.056	0.300	L-phenylalanine, phenylpyruvic acid
7	Fructose and mannose metabolism	3/17	0.068	0.251	Alpha-D-glucose, D-mannitol, Beta-D-fructose 6-phosphate
8	Starch and sucrose metabolism	3/18	0.078	0.266	Sucrose, alpha-D-glucose, beta-D-fructose 6-phosphate
9	Alanine, aspartate and glutamate metabolism	3/20	0.101	0.025	Pyruvic acid, fumaric acid, argininosuccinic acid
10	Sphingolipid metabolism	2/11	0.127	0.314	L-serine, sphinganine
11	Arginine and proline metabolism	4/37	0.156	0.304	L-arginine, ornithine, fumaric acid, argininosuccinic acid
12	Glycine, serine and threonine metabolism	3/26	0.182	0.190	Pyruvic acid, L-serine, L-tryptophan
13	Glyoxylate and dicarboxylate metabolism	2/14	0.189	0.207	Citric acid, *cis*-aconitic acid
14	Tryptophan metabolism	3/27	0.197	0.231	L-tryptophan, anthranilic acid, kynurenine
15	Galactose metabolism	2/17	0.254	0.026	Sucrose, alpha-D-glucose
16	Pentose phosphate pathway	2/18	0.276	0.152	Sucrose, alpha-D-glucose
17	Lipoic acid metabolism	1/6	0.299	0.000	DL-alpha-lipoic acid
18	Pyruvate metabolism	2/23	0.384	0.259	Pyruvic acid, *S*-acetyldihydrolipoamide-E
19	Glutathione metabolism	2/23	0.384	0.429	Glutathione, ornithine
20	Valine, leucine and isoleucine biosynthesis	2/24	0.405	0.020	Pyruvic acid, L-isoleucine
21	Amino sugar and nucleotide sugar metabolism	2/24	0.405	0.159	Alpha-D-glucose, beta-D-fructose 6-phosphate
22	Biosynthesis of unsaturated fatty acids	3/42	0.437	0.000	Arachidonic acid, linoleic acid, alpha-linolenic acid
23	Cyanoamino acid metabolism	1/10	0.447	0.000	L-serine
24	Purine metabolism	4/60	0.455	0.021	Adenine, hypoxanthine, xanthine, cAMP
25	Methane metabolism	1/11	0.479	0.167	L-serine
26	Riboflavin metabolism	1/11	0.479	0.276	Riboflavin
27	Terpenoid backbone biosynthesis	1/15	0.590	0.000	Geranylgeranyl PP
28	Valine, leucine and isoleucine degradation	1/16	0.614	0.000	L-histidine
29	Pantothenate and CoA biosynthesis	1/16	0.614	0.000	Pyruvic acid
30	Histidine metabolism	1/16	0.614	0.071	L-histidine
31	Butanoate metabolism	1/17	0.636	0.000	Pyruvic acid
32	Tyrosine metabolism	1/19	0.678	0.000	L-tyrosine

### Dynamic Metabolites Changes in Enrichment Pathways Involved in Heat Stress

We questioned whether the content of diverse metabolites in a certain pathway fluctuated when the mycelia of *P. ostreatus* encounter heat stress and if high temperature can affect a certain pathway in the mycelia; thus, we further analyzed the metabolite dynamics based on the pathway enrichment results in each group and the normalized data of the differential metabolites. Here, the upregulated metabolic pathways under heat stress were amino acid and vitamin metabolism, glycolysis, and the tricarboxylic acid cycle, while the downregulated metabolic pathways included unsaturated fatty acid and nucleotide metabolism.

**FIGURE 3:**
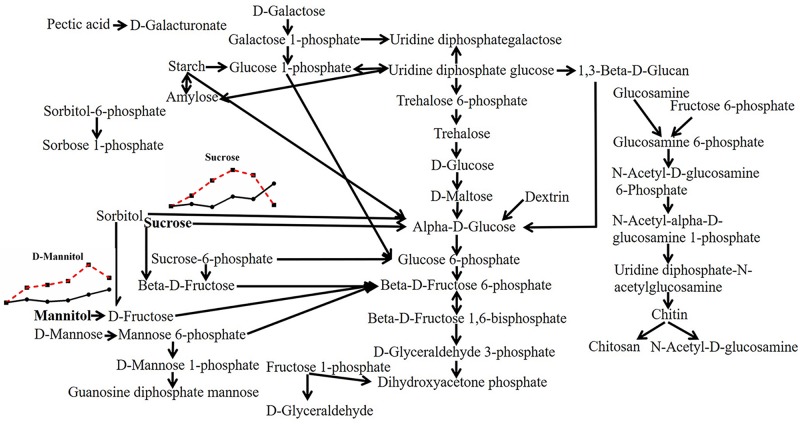
Metabolic pathway of fungal saccharides and changes in differential metabolites in *P. ostreatus* under heat stress. The differential substances are shown in bold letters. The change in metabolites with temperature is represented by a line chart (average), in which the 28°C group is represented by a solid black line with black dots, and the 40°C group is represented by a red dotted line with black squares; the dots or squares indicate 0, 6, 12, 24, 48, and 48 + 6 h. The same scheme is used below.

To explain the response of mycelia to heat stress in more detail, we first made a diagram of all substances involved in saccharides metabolism according to the data of model fungus *S. cerevisiae* in the MetaboAnalyst 4.0 database, which mainly consisted of starch, sucrose, dextrin, trehalose, maltose, fructose, chitosan, mannose and galactose. However, different from the results of the total carbohydrate content detected before (Supplementary Figure S1E), there were few kinds of saccharides that changed with heat stress. Only sucrose (with the peak change value of 1.914-fold under heat stress; multiple changes below were expressed in the same way) and mannitol (1.961-fold) increased with continued heat stress and decreased correspondingly when mycelia resumed growth (Figure 3), which may attribute to the extracon medium (methanol) does not extract high molecular weight compounds such as the polysaccharides. Thus, the experiments just detected the low molecular weight saccharides, and the results showed that heat stress had little effect on low molecular weight saccharides, even the trehalose, a disaccharide act as stress protectant to stabilize cell membranes and protect biological structures (Lei et al., 2019), has not changed significantly here.

**FIGURE 4:**
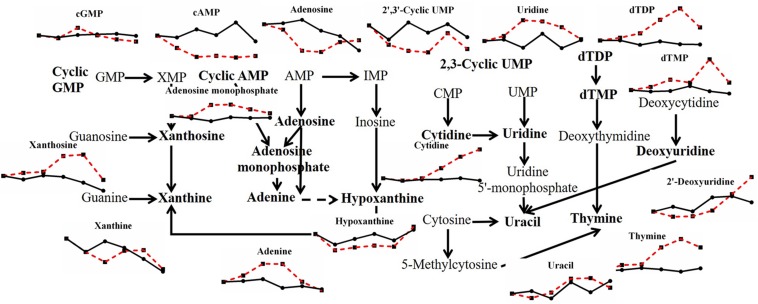
Metabolic pathway of fungal nucleotides and changes in differential metabolites in *P. ostreatus* under heat stress.

In contrast to the variation of saccharides, there were many kinds of nucleotide changed with heat stress in *P. ostreatus* (Figure 4). Almost half of the substances belonging to purine metabolites changed with high temperature. Xanthosine, adenosine monophosphate and adenine increased by 2.154-fold, 2.118-fold, and 2.199-fold, respectively. cAMP, adenosine and hypoxanthine decreased by 0.111-fold, 0.436-fold, and 0.666-fold, respectively. cGMP exhibited a 1.604-fold increase first and then decreased, while xanthine presented the opposite trend, with a 0.602-fold minimal change and a 1.257-fold maximal change. For pyrimidine metabolism, 2′,3′-cyclic UMP (0.422-fold) and 2′-deoxyuridine (0.660-fold) decreased, and cytidine (2.359-fold), uridine (1.775-fold), uracil (1.393-fold), dTDP (2.785-fold), dTMP (2.869-fold), and thymine (3.546-fold) increased under high temperature. Among these, the changed contents of cAMP, cGMP and 2′,3′-cyclic UMP may indicate a participation of cell signals in heat stress; and the changes of adenine, uracil, dTDP, dTMP and thymine may imply an increase in nucleotide decomposition. This may be the result of thermal damage, since it has been reported that heat stress can induce DNA fragment in two *Pleurotus* species after incubating at 42°C for 2 h (Song et al., 2014).

**FIGURE 5:**
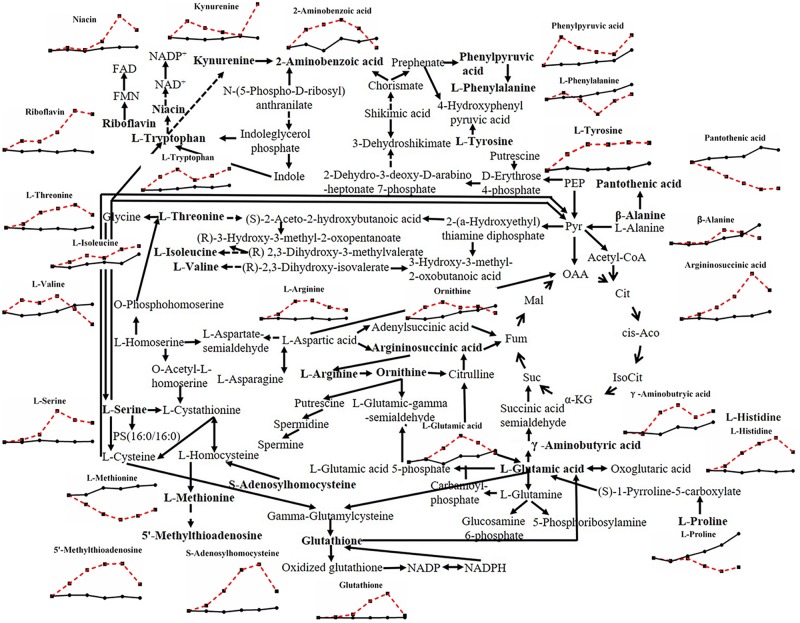
Metabolic pathway of fungal amino acids and changes in differential metabolites in *P. ostreatus* under heat stress.

Most kinds of amino acids varied under heat stress in this study (Figure 5). Amino acids in filamentous fungi can be divided into five groups according to their sources (Xing et al., 2010): those produced from the cycling of tricarboxylic acids, including L-aspartic acid, L-glutamic acid, L-glutamine, and L-lysine; those produced from glycolysis, including L-cysteine, L-phenylalanine, L-tyrosine, L-tryptophan, L-alanine, L-glycine, L-serine, L-valine, L-leucine, and L-isoleucine; those derived from glutamic acid, including L-arginine, L-proline, L-ornithine, and L-citrulline; those derived from L-aspartic acid, including L-cysteine, L-methionine, L-threonine, L-asparagine, L-homoserine; and L-histidine derived from imidazole phosphoglycerol. Almost all kinds of amino acids above and related metabolites showed an increased trend after heat stress, except for L-phenylalanine, L-proline, and L-methionine, which corresponded to the increase in glycolysis and the tricarboxylic acid cycle under heat stress (data not shown). Amino acids are one of the key biochemical precursors (Kistler and Broz, 2015), combined with the fact that heat stress makes total protein content of mycelia reduced significantly (Supplementary Figure S1D), it means that heat stress can lead to the continuous decomposition of protein, thus amino acids continuously changed with heat stress, however, the effects of amino acids on stress defense are rarely reported.

**FIGURE 6:**
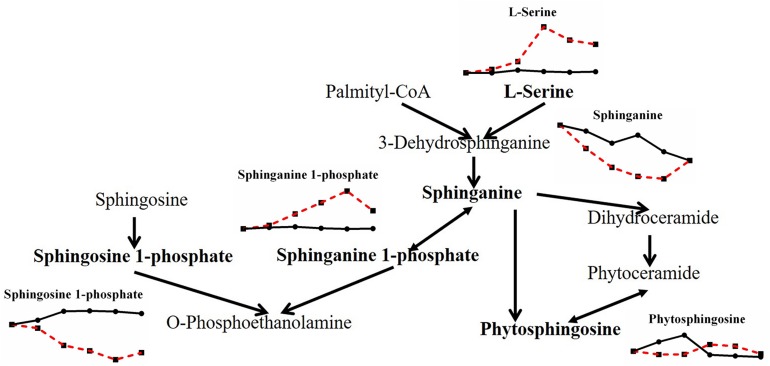
Metabolic pathway of fungal sphingolipids and changes in differential metabolites in *P. ostreatus* under heat stress.

**FIGURE 7:**
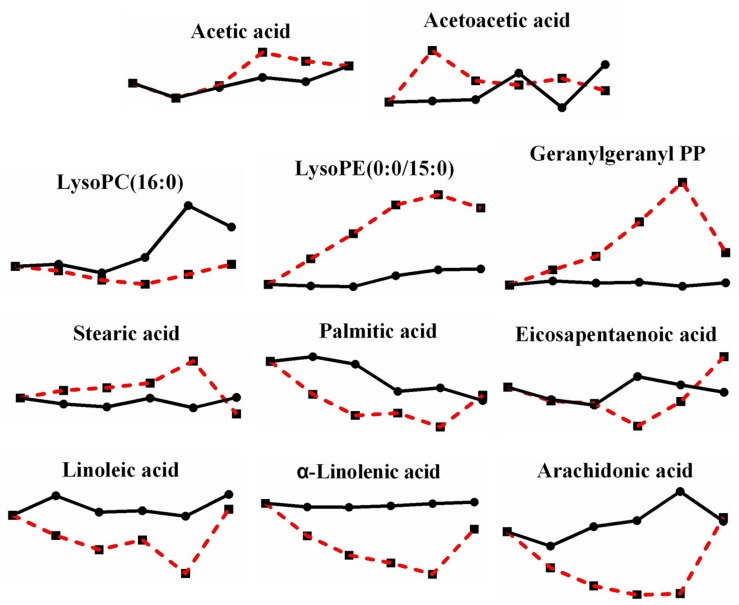
Changes in differential metabolites related to lipid metabolism in *P. ostreatus* under heat stress.

When the effects of heat stress on lipid metabolism in *P. ostreatus* were observed, sphingolipid metabolism showed unknown metabolic trends (Figure 6). Both L-serine (7.816-fold) and sphinganine 1-phosphate (2.607-fold) increased, while both sphinganine (0.205-fold) and sphingosine 1-phosphate (0.219-fold) decreased; phytosphingosine was reduced first (0.343-fold) and then increased (2.148-fold). Geranylgeranyl PP (terpenoid backbone biosynthesis) increased greatly by 9.610-fold, and LysoPC (16:0) and LysoPE (0:0/15:0), involved in glycerophospholipid metabolism, decreased 0.330-fold and increased 6.137-fold, respectively (Figure 7). The metabolites involved in the biosynthesis of unsaturated fatty acids showed a substantial decrease after heat stress, except for stearic acid (1.835-fold) (Figure 7), which may be the reason for the increase in the tricarboxylic acid cycle. Acetic acid and acetoacetic acid, related to acetyl CoA, increased by 1.295-fold and 1.731-fold, respectively (Figure 7). The significantly decreased sphinganine, sphingosine 1-phosphate and LysoPC under heat stress may indicate that the cell membrane is damaged by heat stress, combining that the malondialdehyde (MDA) level significantly increased under heat stress (Lei et al., 2019).

**FIGURE 8:**
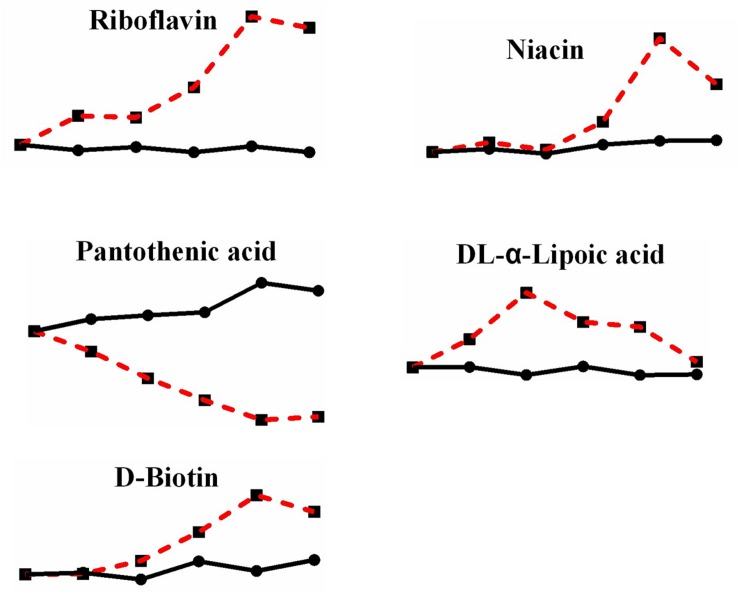
Changes in differential metabolites related to vitamins in *P. ostreatus* under heat stress.

In addition, although not many kinds, there are still some vitamins underwent a change after heat stress (Figure 8). Riboflavin, which is vitamin B2 and exists in the body as flavin mononucleotide (FMN) and flavin adenine dinucleotide (FAD), increased by 4.211-fold; niacin (vitamin B3), a precursor of nicotinamide adenine dinucleotide (NAD) and nicotinamide adenine dinucleotide phosphate (NADP), increased by 2.274-fold; pantothenic acid (vitamin B5), a component of coenzyme A, decreased by 0.202-fold; DL-α-lipoic acid (vitamin B analog), which plays a role in transacylation, increased by 3.920-fold; and D-biotin (vitamin H), which acts as a carboxyl carrier, increased by 1.743-fold. Although we did not detect the changes of NAD or NADP, yet there is significant change in niacin, which also indicate the change of reduction force under heat stress.

**FIGURE 9:**
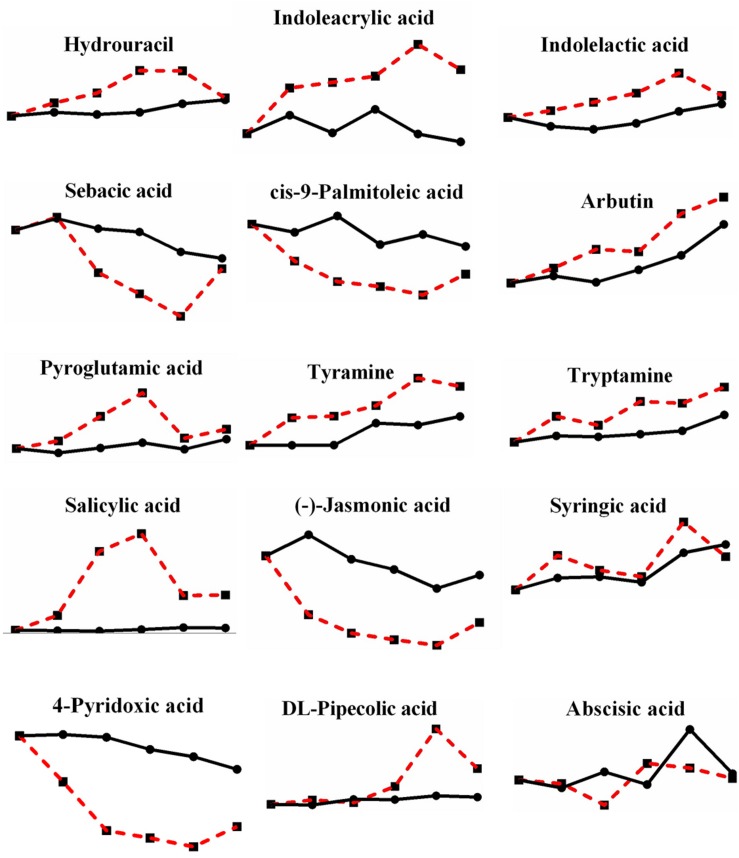
Changes in differential metabolites enriched beyond the pathways in *P. ostreatus* under heat stress.

Moreover, there were still 15 metabolites that were not enriched in their pathways (Figure 9): hydrouracil (2.297-fold increase), indoleacrylic acid (3.144-fold increase), indolelactic acid (1.486-fold increase), sebacic acid (0.248-fold decrease), *cis*-9-palmitoleic acid (0.129-fold decrease), arbutin (2.070-fold increase), pyroglutamic acid (1.837-fold increase), tyramine (1.727-fold increase), tryptamine (2.047-fold increase), salicylic acid (28.794-fold increase), (-)-jasmonic acid (0.118-fold decrease), syringic acid (1.380-fold increase), 4-pyridoxic acid (0.118-fold decrease), DL-pipecolic acid (2.957-fold increase), and abscisic acid (fluctuated between 0.559 and 1.337). Some metabolites may not be in the fungus database, and some were well known as plant anti-stress responses, especially salicylic acid, with the second largest change multiple, reaching up to 28.794-fold, among all metabolites changed during high-temperature stress. In fact, salicylic acid and syringic acid have been reported as a kind of phenolic substance in *P. ostreatus* fruiting bodies, and phenolic compounds are famous for their antioxidant property (Gasecka et al., 2016), therefore, mushrooms containing polyphenols are also popular as nutritional products (Vamanu, 2019). The increase of polyphenols here under heat stress also suggests a possible heat defense mechanism of mushroom, yet, additional studies and discussion of the performance of these metabolites under heat stress are necessary.

### Metabolic Network Construction of *P. ostreatus* Under Heat Stress

**FIGURE 10:**
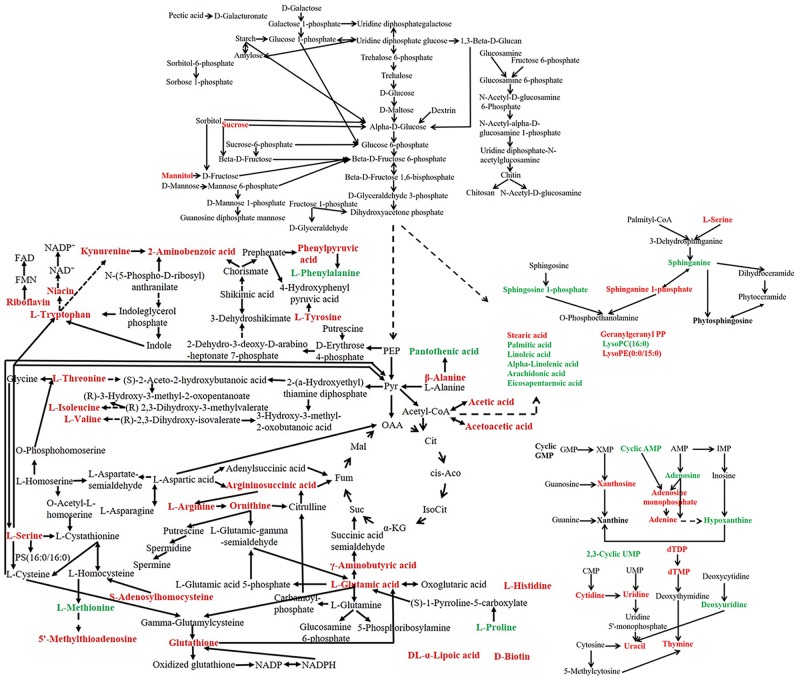
Metabolic network of *P. ostreatus* under heat treatment. The red font indicates an increased content of the substance under heat stress, while the green font indicates a decreased content after heat stress.

A metabolic pathway network was further constructed to show the interactions among the metabolites based on the metabolic pathways changed with heat stress (Figure 10). Overall, heat stress enhanced the degradation of unsaturated fatty acids and nucleotides, increased the content of amino acids and vitamins in *P. ostreatus*.

### Time Course Change of *P. ostreatus* Metabolites to Continuous Heat Stress

**FIGURE 11:**
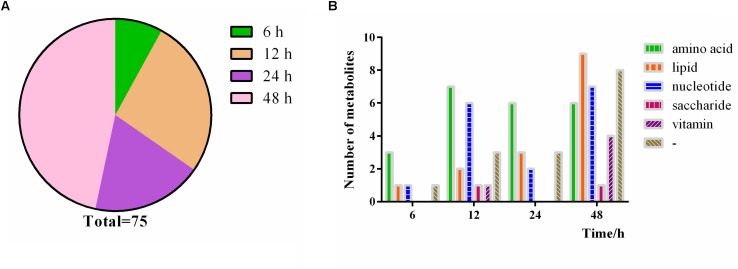
Time course change of *P. ostreatus* metabolites to continuous heat stress. **(A)** The proportion of the occurrence time of the maximum change multiple of the metabolites; **(B)** number of metabolites significantly changed in different heat treat times. The terms “amino acid,” “lipid,” “nucleotide,” “vitamin,” “saccharides,” and “-” in “Metabolic pathway” represent amino acid metabolism, lipid metabolism, nucleotide metabolism, vitamin metabolism, saccharides metabolism, and no pathway, respectively.

During heat treatment, substances changes start at different times, thus enabling a dynamic change process with the continuous heat stress. We sorted the metabolites based on the time when the maximum change multiple occurs under heat stress and classified them in Figure 11 and Supplementary Table S1. A total of 75 differential metabolites were used for pathway analysis. Among the 75 differential metabolites, 6 metabolites reach their largest change multiples at 6 h, which is the smallest proportion; 20 and 14 metabolites meet their largest change multiples at 12 h and 24 h, respectively; and 35 metabolites meet their largest change multiples at 48 h, which occupy the majority of metabolites (Figure 11A). The data indicates that the more kinds of substances are affected with the extension of heat stress time, as expected. Moreover, when analyzing the time course change of *P. ostreatus*, the changes of amino acids are the most obvious after 6 h heat stress, and both amino acids and nucleotides show a stronger change at 12 h than at other time points. The changes of amino acids are still obvious after 24 h heat stress, and all metabolites change strongly under heat stress at 48 h (Figure 11B). On the other hand, the results show that amino acids are susceptible to heat stress, nucleotides are affected obviously by heat stress at 12 and 48 h, and the influence degree of heat stress on lipids is gradually expanded, saccharide and vitamin just have a maximum change multiple at 12 and 48 h respectively owing to fewer kinds of saccharide and vitamin are affected by heat stress.

## Discussion

In this study, we first identified the dynamic alteration of *P. ostreatus* mycelia metabolites during 48 h of heat stress, thereby determining the intracellular metabolic changes that occurred in *P. ostreatus* mycelia in response to heat stress, which mainly included enhanced unsaturated fatty acid and nucleotide degradation, increased content of amino acid and vitamin and increased synthesis of some stress-resistant substances. This is the first study to explain the mechanism of the heat stress response of *P. ostreatus* mycelia from a metabolic perspective; it also lays a foundation for the study of heat tolerance in *P. ostreatus* and other edible and medicinal fungi.

Among the metabolites, we note that riboflavin and niacin, related to the substances with electron transport function (FMN, FAD, NAD, NADP), increased under heat stress, which may indicate that electron transport ability and redox state of cells are affected by heat stress. And they may be the key metabolites helping *P. ostreatus* resist stress. Additionally, the alteration of glutathione under heat stress may be important. Glutathione exhibited the greatest change multiple among all differential metabolites. Because of the antioxidant properties of glutathione, we confirmed the self-resistance of *P. ostreatus* to heat stress; the complete detailed mechanism of that resistance requires further study. As we known, heat stress can cause the outbreak of ROSs, and further damage to mycelia (Liu et al., 2018b; Lei et al., 2019), if we can apply the above-mentioned substances to regulate the level of redox when the mycelia may suffer from heat stress, it may be able to remove part of ROS, thus causing lower damage to the mycelia and stabilizing the bioactive components in the mycelia.

Edible mushroom polysaccharides have been widely studied for their pharmacological activities, especially in *P. ostreatus* (Pandya et al., 2019). Moreover, a report (Tan et al., 2018) told that heat stress can enhanced the production of polysaccharides in *G. lucidum* after the mushroom was harvest and treated with 42°C for 2 h, and that resulted in the highest polysaccharide yield of 10.50% (45.63% higher than that of the control). Due to the limitation of metabonomic method for polysaccharide extraction, we do not get the polysaccharide content in *P. ostreatus* here, but the fact that heat stress makes the total carbohydrate content of mycelia reduced significantly may imply that the mycelia are not the suitable material for obtaining polysaccharide compared with fruiting body. In addition, amino acids are the source of mushroom flavor and the mycelia were reported to have higher amounts of flavor compounds than that in fruiting bodies (Kim et al., 2011). Considering that the content of amino acids here increased generally under heat stress, it will be benefit to applicate the *P. ostreatus* mycelia under moderate heat treatment in food and flavor industry.

The continued decline in the content of cAMP with heat stress attracts our attention. In fact, cAMP has been reported in many studies to act as a signal molecule, in addition to acting as an intermediate metabolite involved in metabolism. In these studies, cAMP is shown to be involved in the cAMP/PKA signaling pathway, in which elevated cAMP activated PKA, and then, the PKA pathway promotes the activation of the Efg1 transcription factor, inducing the expression of downstream genes (Saputo et al., 2014; Parrino et al., 2017). In *S. cerevisiae*, cAMP signaling is the downstream of glucose-sensing G proteins; thus, the cAMP-PKA pathway is considered to be associated with fungal secondary metabolism (Choi and Xu, 2010). Similarly, the downregulated Efg1-mediated cAMP/PKA pathway may activate the alcohol dehydrogenase and glycolytic enzymes in *Candida albicans* (Ku et al., 2017), and the cAMP signaling pathways were also reported to regulate hyphal growth coordinately with the tricarboxylic acid cycle in *C. albicans* (Tao et al., 2017). Moreover, the cAMP-PKA pathway has also been shown to regulate stress responses, as a mutant with reduced intracellular levels of cAMP showed increased heat resistance (Choi and Xu, 2010), which may agree with the results in this study and indicate the role of cAMP as a signaling molecule. Furthermore, the results also suggested that there is an interaction between metabolism and signaling to cope with heat stress.

Perhaps there are similar signaling materials, such as cAMP, waiting to be discovered in the heat stress effect; however, interestingly, some substances that were not enriched based on the fungus database changed significantly to heat stress. These substances, including salicylic acid, jasmonic acid, syringic acid, DL-pipecolic acid, and abscisic acid, all play important roles in the adaptation of plants to environmental stress (Tokmak et al., 2015; Wang et al., 2015; Sirhindi et al., 2016; Martínez-Medina et al., 2017; Pérez-García et al., 2017). Moreover, *Trichoderma* (Martínez-Medina et al., 2017) regulated defenses by shifting from priming salicylic acid to jasmonic acid to protect tomato against the root knot nematode *Meloidogyne incognita*, providing additional evidence for the production of salicylic acid and jasmonic acid by fungi. Salicylic acid, jasmonic acid and abscisic acid are all hormone signals that play major roles in mediating plant defense responses against pathogens and abiotic stresses (Verma et al., 2016). Among those, salicylic acid and jasmonic acid were reported to play significant roles in regulating plant defense responses against pathogens and pests (Bari and Jones, 2009); however, these phytohormones have also been reported to play a role in fungal growth and secondary metabolism in recent years (Ren et al., 2010; Liu et al., 2018a), and both have significant thermal changes in this research, especially salicylic acid. In addition, abscisic acid is responsible for plant defense against abiotic stresses (Bari and Jones, 2009), and heat stress caused a significant increase in the endogenous abscisic acid content in plants (Abdelrahman et al., 2017), although it fluctuated in *P. ostreatus* for unknown reasons.

In fact, although we have found a series of substances that changed under heat stress through metabolome, we only classify them into several metabolites such as saccharides, unsaturated fatty acid, nucleotide, amino acid and vitamin; and even if we confirmed the change trend of these substances in 48 h of heat stress, the detailed metabolic relationship between these substances is uncertain; moreover, to better understand the response of mushroom to heat stress and the mechanism or method of stress-resistance, more studies should be conducted on the metabolic pathways, however we have not shown the possible upstream and downstream relationships between metabolites in the metabolic pathways, which needs other study.

From these results, we found that metabolic changes occurred in *P. ostreatus* mycelia to adapt to heat stress. We concurrently identified multiple levels of alteration under heat stress and that there are interactions between different levels (such as signal and metabolism), which reminds us to explore heat resistance from a broader perspective, such as the crosstalk within different metabolic pathways and the detailed connections between different levels. Moreover, even though the changes of metabolites in *P. ostreatus* mycelia reflect the response and defense mechanisms of mycelia to heat stress, yet considering the biological values of mushrooms in biopharmaceutical and food industry (Gu and Sivam, 2006; Kim et al., 2011; Ma et al., 2018; Zhu et al., 2019), the increase of some key compounds such as amino acids and polyphenol in *P. ostreatus* under heat stress may be a powerful measures to obtain the useful ingredients and improve the efficiency to the related research and industry, thus may be a new research fields in the future.

## Data Availability Statement

All datasets generated for this study are included in the article/Supplementary Material.

## Author Contributions

ZY and MZ conceived, designed, and performed the experiments, analyzed the data, and wrote and revised the manuscript. JZ conceived and designed the experiments. XW revised the manuscript.

## Conflict of Interest

The authors declare that the research was conducted in the absence of any commercial or financial relationships that could be construed as a potential conflict of interest.
