# Supplementary figures and images for: Metabolic Response of Pleurotus ostreatus to Continuous Heat Stress
Source: Front Microbiol. 2020 Jan 21;10:3148. doi: 10.3389/fmicb.2019.03148 (PMC6990131; doi:10.3389/fmicb.2019.03148)

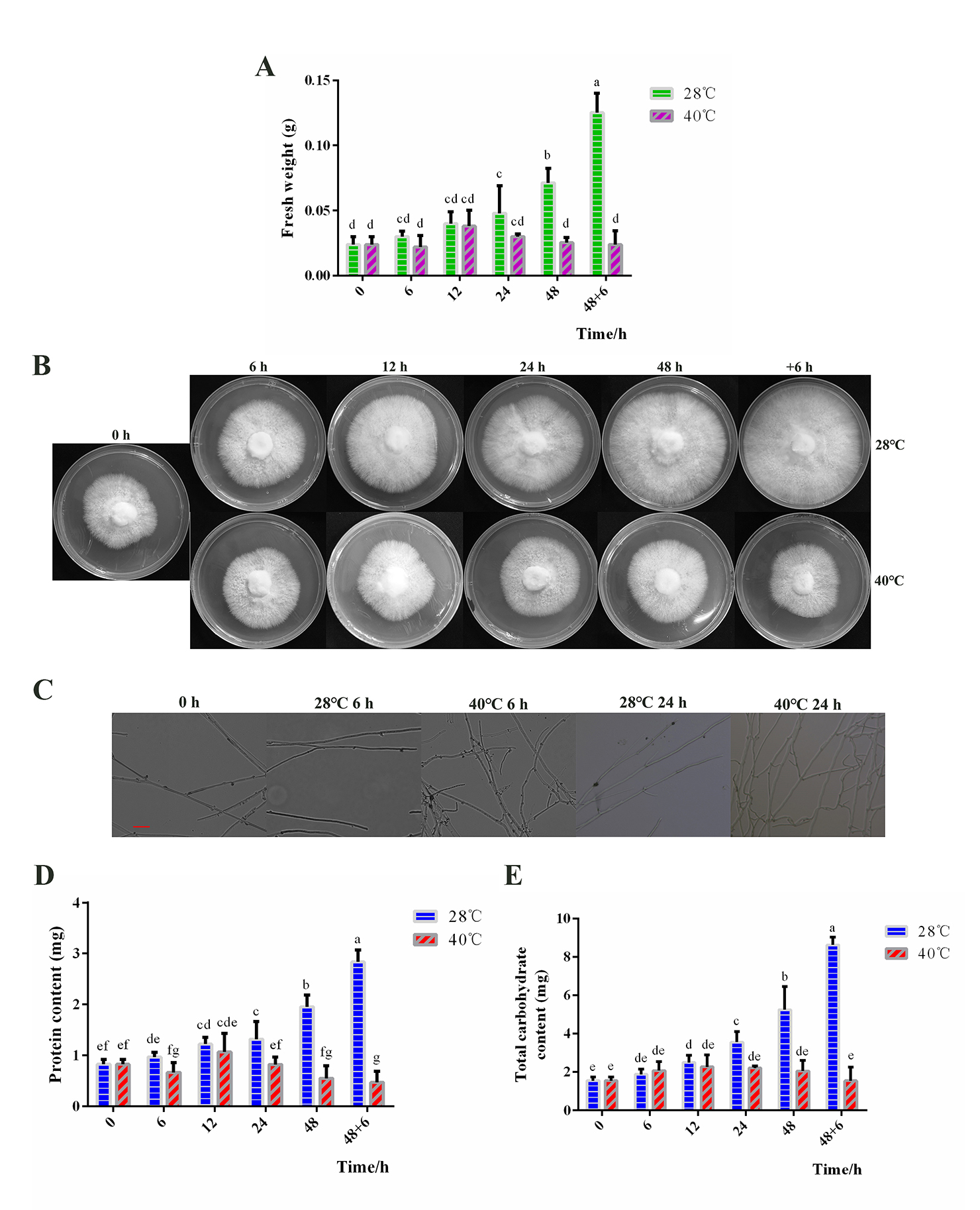

Supplement: Supplementary file 1 [file Image_1.JPEG]
