# Supplementary material for: Metabolic Response of Pleurotus ostreatus to Continuous Heat Stress
Source: Front Microbiol. 2020 Jan 21;10:3148. doi: 10.3389/fmicb.2019.03148 (PMC6990131; doi:10.3389/fmicb.2019.03148)
Supplement: Supplementary file 2 [file Table_1.DOCX]

Metabolic Response of *Pleurotus ostreatus* to Continuous Heat Stress

Supplementary Material

**Supplementary** **Figure 1.** The effect of heat stress on the basic growth phenotype of *P. ostreatus*. (A) the fresh weight change, n=3; (B) the macroscopic alteraons in the mycelia of *P. ostreatus* under heat stress, n=3; (C) the microscopic alteraons in the mycelia of *P. ostreatus* after heat treatment 6 h and 24 h, scale bar =100 μM; (D) the effect of heat stress on the total protein content of *P. ostreatus*, n=6; (E) the effect of heat stress on the total carbohydrate content of *P. ostreatus*, n=6. Data expressed as mean ± SD. P < 0.05 by Duncan’s multiple range test. 48 h+6 h means heat treated 48 h and then recovered 6 h.

**Supplementary Table 1.** Time course response of metabolites of *P. ostreatus* to continuous heat stress.

| Heat treatment time/h | Metabolites | Metabolic pathway | Heat treatment time/h | Metabolites | Metabolic pathway |
| --- | --- | --- | --- | --- | --- |
| 6 | Phenylpyruvic acid | amino acid | 24 | Pyroglutamic acid | - |
|  | L-Valine | amino acid |  | Hydrouracil | - |
|  | Kynurenine | amino acid | 48 | D-Mannitol | polysaccharide |
|  | Acetoacetic acid | lipid |  | Riboflavin | vitamin |
|  | 2'-Deoxyuridine | nucleotide |  | D-Biotin | vitamin |
|  | Syringic acid | - |  | Niacin | vitamin |
| 12 | Arbutin | - |  | Pantothenic acid | vitamin |
|  | Abscisic acid | - |  | 4-Pyridoxic acid | - |
|  | Salicylic acid | - |  | cis-9-Palmitoleic acid | - |
|  | Phytosphingosine | lipid |  | Sebacic acid | - |
|  | LysoPE(0:0/15:0) | lipid |  | Indoleacrylic acid | - |
|  | DL-alpha-Lipoic acid | vitamin |  | Indolelactic acid | - |
|  | Sucrose | polysaccharide |  | Tyramine | - |
|  | 2’,3’-Cyclic UMP | nucleotide |  | DL-Pipecolic acid | - |
|  | Xanthine | nucleotide |  | (-)-Jasmonic acid | - |
|  | Uridine | nucleotide |  | Sphingosine 1-phosphate | lipid |
|  | cGMP | nucleotide |  | Geranylgeranyl PP | lipid |
|  | Adenosine monophosphate | nucleotide |  | Palmitic acid | lipid |
|  | Adenosine | nucleotide |  | Stearic acid | lipid |
|  | L-Glutamate | amino acid |  | Arachidonic acid | lipid |
|  | Gamma-Aminobutyric acid | amino acid |  | Sphinganine 1-phosphate | lipid |
|  | Ornithine | amino acid |  | LysoPC(16:0) | lipid |
|  | L-Arginine | amino acid |  | Linoleic acid | lipid |
|  | Anthranilic acid | amino acid |  | Alpha-Linolenic acid | lipid |
|  | L-Tyrosine | amino acid |  | dTMP | nucleotide |
|  | L-Tryptophan | amino acid |  | Xanthosine | nucleotide |
| 24 | Acetic acid | lipid |  | cAMP | nucleotide |
|  | Sphinganine | lipid |  | Uracil | nucleotide |
|  | Eicosapentaenoic acid | lipid |  | Thymine | nucleotide |
|  | Hypoxanthine | nucleotide |  | dTDP | nucleotide |
|  | Adenine | nucleotide |  | Cytidine | nucleotide |
|  | L-Serine | amino acid |  | L-Proline | amino acid |
|  | L-Methionine | amino acid |  | Argininosuccinic acid | amino acid |
|  | Beta-Alanine | amino acid |  | L-Histidine | amino acid |
|  | L-Phenylalanine | amino acid |  | L-Threonine | amino acid |
|  | 5'-Methylthioadenosine | amino acid |  | L-Isoleucine | amino acid |
|  | Glutathione | amino acid |  | S-Adenosylhomocysteine | amino acid |
|  | Tryptamine | - |  |  |  |

Note: the terms “amino acid”, “lipid”, “nucleotide”, “vitamin”, “polysaccharide”, and “-” in “Metabolic pathway” represent amino acid metabolism, lipid metabolism, nucleotide metabolism, vitamin metabolism, polysaccharide metabolism, and no pathway, respectively.
